# Dr. Andrew Benjamin Knisley

**Published:** 1917-03

**Authors:** 


**Dr. Andrew Benjamin Knisley,** Victoria of Toronto 1885, died at his home in Buffalo Jan. 10, aged 58.

